# Induction of the CtsR regulon improves Xylanase production in *Bacillus subtilis*

**DOI:** 10.1186/s12934-023-02239-3

**Published:** 2023-11-09

**Authors:** Biwen Wang, Frans van der Kloet, Leendert W. Hamoen

**Affiliations:** Swammerdam Institute for Life Sciences, University of Amsterdam, Science Park 904, C3.108, 1098 XH Amsterdam, The Netherlands

**Keywords:** *Bacillus*, Xylanase, Secretion, RNA-seq, *ctsR*, Clp chaperones

## Abstract

**Background:**

The bacterium *Bacillus subtilis* is extensively used for the commercial production of enzymes due to its efficient protein secretion capacity. However, the efficiency of secretion varies greatly between enzymes, and despite many years of research, optimization of enzyme production is still largely a matter of trial-and-error. Genome-wide transcriptome analysis seems a useful tool to identify relevant secretion bottlenecks, yet to this day, only a limited number of transcriptome studies have been published that focus on enzyme secretion in *B. subtilis*. Here, we examined the effect of high-level expression of the commercially important enzyme endo-1,4-β-xylanase XynA on the *B. subtilis* transcriptome using RNA-seq.

**Results:**

Using the novel gene-set analysis tool GINtool, we found a reduced activity of the CtsR regulon when XynA was overproduced. This regulon comprises several protein chaperone genes, including *clpC*, *clpE* and *clpX*, and is controlled by transcriptional repression. CtsR levels are directly controlled by regulated proteolysis, involving ClpC and its cognate protease ClpP. When we abolished this negative feedback, by inactivating the repressor CtsR, the XynA production increased by 25%.

**Conclusions:**

Overproduction of enzymes can reduce the pool of Clp protein chaperones in *B. subtilis,* presumably due to negative feedback regulation. Breaking this feedback can improve enzyme production yields. Considering the conserved nature of Clp chaperones and their regulation, this method might benefit high-yield enzyme production in other organisms.

**Supplementary Information:**

The online version contains supplementary material available at 10.1186/s12934-023-02239-3.

## Background

Owing to its superior enzyme secretion capacity and safe use, the bacterium *Bacillus subtilis* and its close relatives are extensively employed for the industrial-scale production of enzymes for the detergent, food, paper and pharmaceuticals industries [1–4]. Many genetic strategies have been applied to optimize *B. subtilis* strains for the production of heterologous proteins, including the use of strong promoters, optimizing ribosomal binding sequences, and inactivating its main extracellular proteases [5–9]. This is generally followed by screening for optimal secretion signal peptides [10, 11], and sometimes by increasing the level of certain protein chaperones, such as the cytoplasmic chaperone DnaK or the extra-cytoplasmic chaperone PrsA [12, 13]. However, the range of enzymes that can be produced by *B. subtilis* at high levels is still limited and the yields vary largely [1, 14]. Since production bottlenecks are generally enzyme specific, the use of genome-wide transcriptome analyses is a powerful method to identify them. Nevertheless, there are only a limited number of published transcriptome studies that focus on identifying bottlenecks in the production of commercial enzymes by *Bacillus* species [15–20]. One of the main secretion stress markers observed in these studies is the upregulation of the membrane associated quality control proteases HtrA/B [15–18, 21]. However, removing HtrA/B generally lowers the secretion capacity for both native and heterologous proteins [22, 23], and as far as we know, none of these studies has led to modifications resulting in improved secretion. Here, we used RNA-seq analysis to reveal possible limiting steps in the overproduction of the commercially relevant secreted endo-1,4-β-xylanase XynA by *B. subtilis*.

XynA is an endogenous 23 kDa secreted protein that catalyses the hydrolysis of 1,4-β-d-xylosidic linkages in xylans [24, 25]. The enzyme is used in many processes, including bleaching of wood pulp in the paper industry, desizing and bioscouring in the textile industry, improving dough quality in the bakery industry, and as food additive to poultry [26]. We performed RNA-seq on XynA overexpressing cells collected from different growth phases and applied an in-house developed software tool, Gintool, to facilitate gene set enrichment analysis, respecting the directionality of the regulon-gene interactions [27]. This revealed an unexpected downregulation of Clp protein chaperones. Inducing the expression of these chaperones increased the production of XynA by 25%.

## Results

### Conditions for RNA-seq analysis

**Fig. 1:**
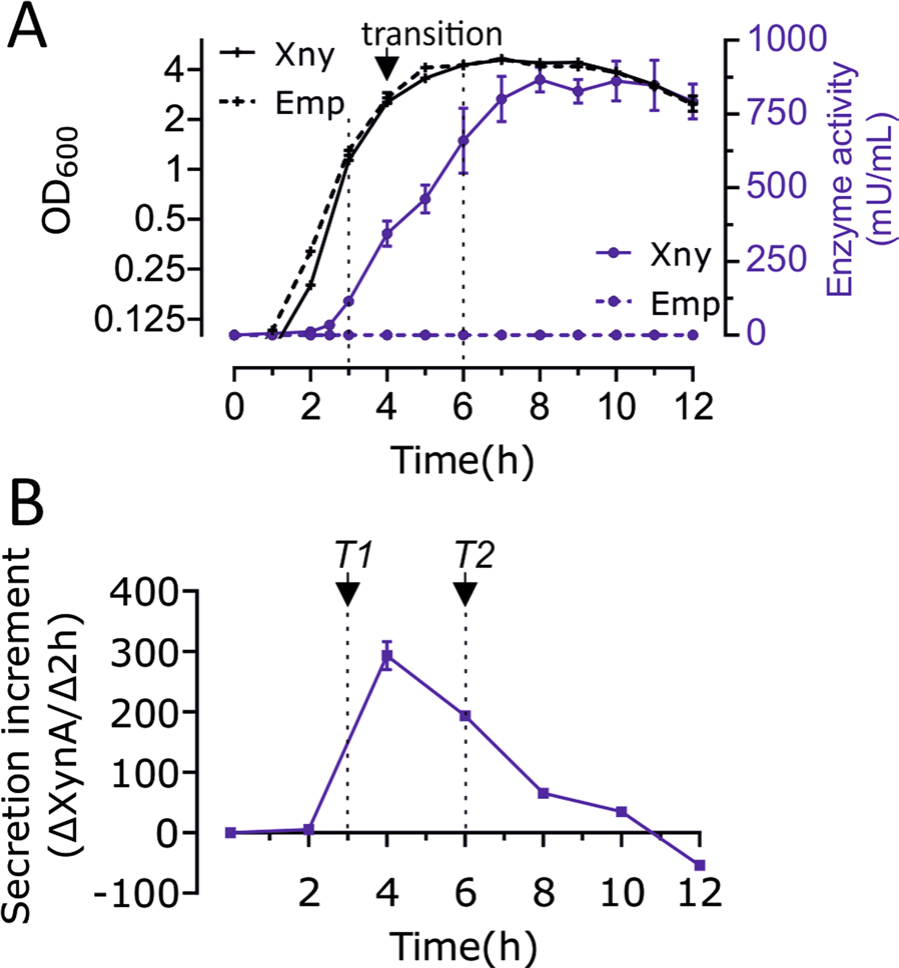
Xylanase production profile in *B. subtilis*. **A** Enzyme activity and growth curve of the BWB09/pCS58 (Xny) and control BWB09/pBW17 (Emp) strains. The arrow indicates the transition from log to stationary phase. **B** Enzyme secretion dynamics during growth, indicated by the enzyme activity change (Δ mU/mL) every 2 h. Sample times for RNA isolation are indicated by arrows (T1 is OD_600_ ~ 0.8 at 3 h, T2 is OD_600_ ~ 4.0 at 6 h)

To ensure that the RNA-seq analysis was performed under relevant conditions, we first measured the accumulation of XynA in the medium during culturing of the *B. subtilis* production strains. The native *xynA* gene was removed by means of a marker-free clean deletion procedure [28], to ensure that the negative control strain does not produce any XynA. Overexpression of XynA was achieved by cloning *xynA* in the multicopy expression plasmid pUB110 behind the strong *amyQ* promoter, and with its own (native) signal peptide [29, 30]. As a negative control we used the same plasmid lacking the *xynA* gene. The strains were grown in nutrient-rich LB medium at 37 °C in the presence of 50 μg/mL kanamycin to maintain the plasmid. Samples were taken at regular time points during growth and the xylanase activity in the medium was measured by a fluorescence-based enzyme activity assay. As shown in Fig. 1a, the overexpression of XynA does not affect the growth rate, and XynA started to accumulate in the medium halfway during the logarithmic growth phase and peaked approximately 1 h after the transition to the stationary phase of growth (Fig. 1b). Production continued for approximately another 6 h. For the RNA-seq analysis, the culture was sampled at 3 h (T1, OD_600_ ~ 0.8) and 6 h (T2, OD_600_ ~ 4).

### Downregulation of the SRP RNA subunit

**Fig. 2:**
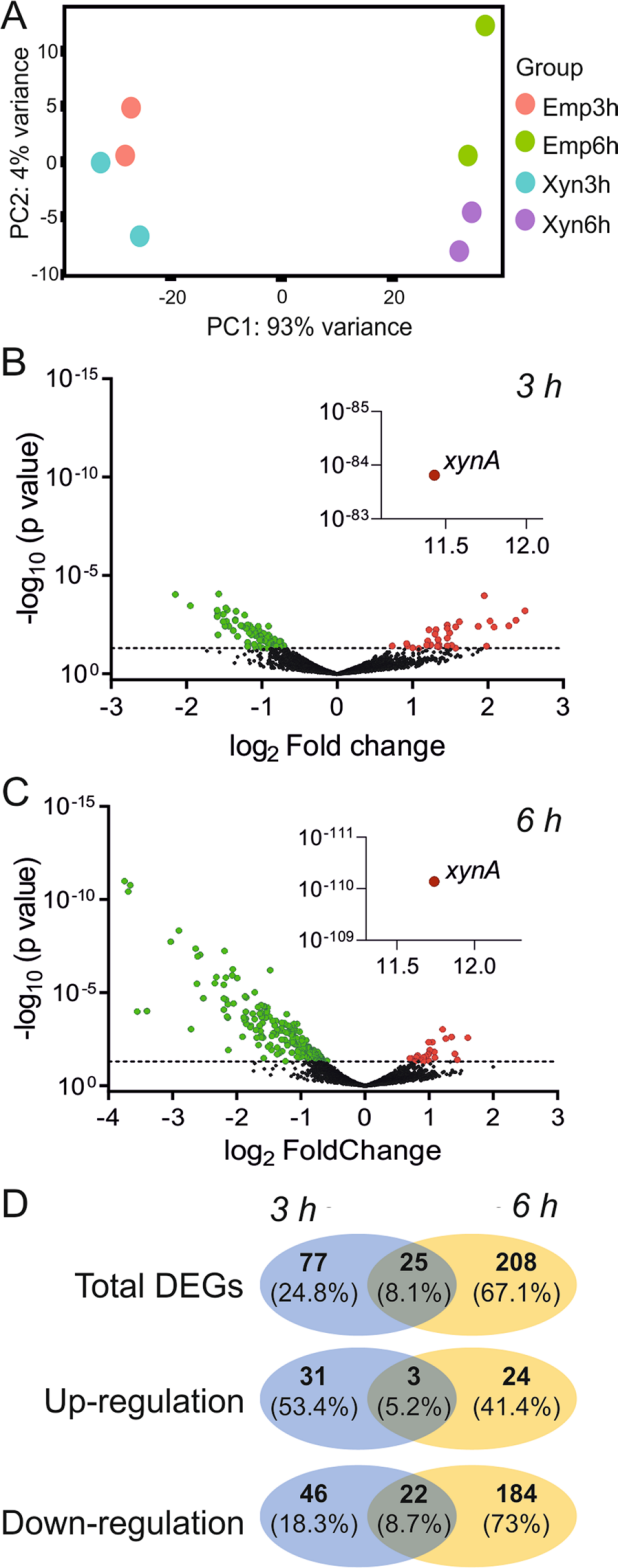
Global comparison of RNA-seq data of XynA overexpressing *B. subtilis* cells. Data from secreting (Xyn) and control strains containing the empty plasmid (Emp) at logarithmic growth (3 h) and stationary growth (6 h) are compared. **A** Principal component analysis (PCA) of the two biological replicates. Emp3h and Emp6h are RNA-seq samples of the control strain containing empty plasmid sampled at 3 and 6 h of growth, while Xyn3h and Xyn6h are RNA-seq samples of the Xyna secreting strain. The largest sample variation is caused by different growth phases (PC1, 3 h vs. 6 h), and the difference between the timepoints in PC1 is consistent for both the Xyn and Emp samples. **B**, **C** Volcano plots of differentially expressed genes (DEGs) induced by XynA overexpression (BWB09/pXynA vs. BWB09/pEmp) at 3 h and 6 h. The dashed line indicates a *p*-value of 0.05. Significantly up- and downregulated genes (*p*-value < 0.05) are colored in red and green respectively. **D** Venn diagrams with the number of significant up- and downregulated genes (*p*-value < 0.05) affected by the stress of XynA secretion at 3 h (in blue) and 6 h (in yellow)

The RNA-seq data are based on two independent biological replicates. A principal component analysis (PCA) shows that the samples cluster primarily based on sampling time (Fig. 2a), which is not surprising considering the large differences between logarithmic and stationary growth phases in which the 3 h and 6 h samples were collected, respectively. The distribution of up- and downregulated genes is presented in the volcano plots of Fig. 2b, c, and shows that XynA overproduction changes the expression of a substantial number of genes, especially in the stationary phase. When a *p*-value < 0.05 was used as threshold, 102 and 233 differentially expressed genes were identified in the log phase and stationary phase samples, respectively. Only 25 of these genes were affected in both time points (Fig. 2d). To focus on the most relevant genes, we further applied a threshold of twofold change for the 3 h time sample, and < -3 or > 3-fold change for the 6 h sample. These genes are listed in Tables 1 and 2, respectively (for a list of all genes, see Additional file 1: Table S1).

**Table 1:** Genes showing more than twofold expression difference at 3 h growth

FC 3 h	FC 6 h	Gene(s)	Operon	Regulon	Function
2800	3400	*xynA*			Endo-1,4-beta-xylanase
5.6/3	1.4/− 1.0	*yxeK,N,M,O,Q,snaB, sndB*	*yxeK (7)*	CymR 100%↓	Uptake, utilization, detoxification S-(2-succino) cysteine
5.2	1.1	*yvdE*	*yvdE (9)*		Unknown
3.9	1.4	*sndA*	*snaA (12)*	CymR 100%↓	Utilization of S-methyl-cysteine
3.9/3	1.4/− 1.1	*cysH,P,C,sat,ylnD*	*cysH (7)*	CymR 100%↓	Sulfate uptake & reduction
3.1/2.3	1.2/− 1.4	*pyrAA,AB,D,E,F,K*	*pyrR (10)*	PyrR 0%-80%	Pyrimidine biosynthesis
3.0	1.5	*yeeG*			Unknown
3.0	1.1	*tcyP*		CymR 100%↓	Cystine (cysteine dimer) uptake
2.7	1.4	*spoVID*	*spoVID (2)*	SigE 96%↑	Spore coat assembly
2.6	1.8	*dppB*	*dppA (5)*	CodY 91%↓	Dipeptide transporter subunit
3	1.3	*nupO*	*nupN (4)*	CodY 91%↓	Uptake of guanosine
3	− 1.1	*putB*	*putB (3)*	PutR 100%↑	Proline dehydrogenase
2.1	1.1	*rhiL*	*rhiL (4)*		Uptake of rhamnose oligosaccharides
2.0	2.1	*leuB*	*ilvB (7)*	CcpA 87%↑	Biosynthesis of leucine
− 4.4	− 8.2	*melR*	*melR (5)*	MelR 100%↑	repressor melibiose utilization operon
− 3.9	− 13.4	*clpE*		CtsR 100%↑	AAA unfoldase
− 3.0	− 2.0	*iseA*		WalR 100%↑	Inhibitor of autolysins
− 3.0/− 3	− 1.7/− 1.2	*rbsA,B,C,D,K,R*	*rbsR (6)*	CcpA 87%↑	Ribose uptake
− 3.0/− 2.4	− 1.4	*yyzE,bglA*	*bglA (2)*		Beta-glucoside utilization
− 3.0/− 2.3	− 1.7/− 1.4	*licA,B,C,H*	*licB (4)*	LicR 100%↓	Lichenan uptake and phosphorylation
− 2.8/− 2.1	− 1.4/− 1.2	*araA,B,D,L,M,N,abfA*	*araA (9)*	AraR 100%↑	Arabinose utilization
− 2.8	− 1.1	*glpD*		GlpP 100%↓	Glycerol-3-phosphate dehydrogenase
− 3	− 1.6	*ywsB*		SigB 100%↓	General stress protein
− 2.4/− 2.1	1.5/1.3	*khtT,U,S*	*khtS (3)*		Potassium ion efflux
− 2.3/− 2.1	− 1.5/− 1.3	*gntK,R*	*gntR (4)*	GntR 100%↑	Gluconate utilization
− 2.3	1.4	*mrgA*	*yusZ (2)*	PerR 100%↑	Protecting against oxidative killing
− 2.3	− 1.2	*ylbP*			Unknown
− 2.3	− 2.7	*yerA*	*yerA (3)*	CcpA 87%↑	Similar to adenine desaminase
− 2.2/− 2.0	− 2.6/− 2.0	*ctsR, mcsA,B,clpC*	*ctsR (6)*	CtsR 100%↑	Class iii heat shock proteins
− 2.2	− 1.8	*yuaF*	*yuaF (3)*	SigW 100%↓	Involved membrane fluidity control
− 2.1	1.3	*manR*		ManR 100%↓	Regulation of mannose utilization
− 2.1/− 2.0	− 1.7/− 1.4	*ganA,P,S*	*ganS (5)*	GanR 100%↑	Uptake of galactotriose
− 2.1	− 1.1	*trpB*	*trpE (6)*	MtrB 0%-13%	Tryptophan synthase subunit
− 2.1	1.1	*katA*		PerR 100%↑	Main catalase
− 2.0	− 1.4	*gmuB*	*gmuB (8)*	GmuR 100%↑	Glucomannan utilization
− 2.0	− 1.0	*uxaC*	*uxaC (10)*	CcpA 87%↑	Glucuronate isomerase

Only genes with *p*-value < 0.05 are shown, and RNAs with unknown functions have not been included in the table. The fold-change (FC) is shown using a linear scale and not as log_2_. FC of the 6 h sample are also listed. Genes in an operon are clustered. In these cases the maximum and minimum FC are indicated. In the operon column the number of genes of the operon is listed between brackets. The regulon column suggests the possible regulator. The percentage shows the probability of regulation indicated by the arrow (up is activation and down is repression). A gene can be part of different regulons, however, only the regulon with the largest percentage of significantly expressed genes is shown. For some regulons the regulation directionality is not present in the Subtiwiki database. This is indicated by 0% for probability of regulation direction and the absence of an arrow

**Table 2:** Genes showing more than twofold upregulation or more than threefold down regulation at 6 h growth

FC 6 h	FC 3 h	Name	Operon	Regulon	Description
3400	2760	*xynA*			Endo-1,4-beta-xylanase
3	1.3	*sr1*		CcpN 100%↓	Small RNA regulation arginine metabolism
2.7	1	*ydjB*			Prophage 3
2.6	2.7	*yuiA*	*yuiA (2)*	TnrA 100%↓	Unknown
2.6	− 1.2	*gapB*	*gapB (2)*	CcpN 100%↓	Glyceraldehyde-3-phosphate dehydrogenase
2.4	− 1	*ribE*	*ribD (5)*	FMN 100%↓	Riboflavin biosynthesis
2.3/2.1	2.0/1.3	*leuB,C*	*ilvB (7)*	CcpA 100%↑	Biosynthesis of leucine
2.1	1.2	*natA*	*natA (2)*	NatR 100%↑	Sodium exporter subunit
2.1	− 1.1	*pckA*		CcpN 100%↓	Phosphoenolpyruvate carboxykinase
2	− 1.2	*frlR*			Regulator sugar amines utilization
− 13.4	− 3.9	*clpE*		CtsR 100%↑	AAA unfoldase
− 12.9/− 8.2	− 4.4/− 1.7	*melA,C,D,E,R*	*melR (5)*	MelR 100%↑	Melibiose utilization
− 10.2/− 3.0	− 1.6/2.0	*sboA,albB,C,D,E,F,G*	*sboA (9)*	ResD 100%↓	Subtilosin (antimicrobial peptide) production
− 7.5/− 3.5	− 1.4/− 2.0	*iolA,G,H,I,J*	*iolA (10)*	IolR 100%↑	Myo-inositol catabolism
− 6.6	1.1	*iolT*		IolR 100%↑	Myo-inositol uptake
− 6.2	1.7	*yebD*			Unknown
− 5.8	− 1.4	*fnr*		ResD 100%↓	Anaerobiosis, overflow metabolism
− 4.8/− 3.1	− 2.0/− 1.2	*yojA,B*	*yojA (2)*		Unknown
− 4.6	− 1.3	*trnB-Arg*			Transfer RNA-Arg
− 4.6/− 4.4	− 1.1/1.0	*ycnI,J,K*	*ycnK (3)*	YcnK 100%↑	Regulation of copper uptake
− 4.6	− 1.0	*ctaA*		ResD 100%↓	Heme biosynthesis
− 4.6	− 1.0	*ydbL*			Unknown
− 4.6/− 3.6	− 1.0/1.0	*qoxA,B,C,D*	*qoxA (4)*	CitB 0%-50%	Cytochrome aa3 quinol oxidase
− 4.5	1.0	*yxaL*	*yxaJ (2)*	Rok 100%↑	Unknown
− 4.4	1.0	*ctaO*		AbrB 100%↑	Heme biosynthesis
− 4.4	− 1.0	*yodA*			Unknown
− 4.4	1.2	*trnB-Leu1*			Transfer RNA-Leucine
− 4.0	1.3	*scr*			4.5S RNA of signal recognition particle (SRP)
− 3.8	1.0	*ydzA*			Unknown
− 3.6	− 1.1	*yozB*			Unknown
− 3.6/− 3.1	− 1.3/− 1.2	*iolR,S*	*iolR (2)*	IolR 100%↑	Regulation of myo-inositol catabolism
− 3.6	1.2	*yoqM*		AbrB 100%↑	SP-beta prophage
− 3.4	− 1.1	*ywzB*	*ywzB (3)*	SigF 75%↓	Unknown
− 3.4	− 1.1	*ytzI*			Unknown
− 3.3	− 1.4	*yxiE*	*bglP (3)*	LicT 100%↓	Possible beta-glucan/-glucoside utilization
− 3.3	1.0	*yfmQ*			Unknown
− 3.2	− 1.7	*yncC*			Unknown
− 3.2/− 3.0	− 1.3/− 1.1	*hemE,H,Y*	*hemE (3)*		Heme biosynthesis
− 3.1	− 1.2	*gabT*	*gabT (2)*	GabR 100%↓	Utilization of gamma-amino butyric acid
− 3.1	1.2	*sunA*	*sunA (5)*	Rok 100%↑	Sublancin (antimicrobial peptide) production
− 3.1	− 1.0	*ytkA*	*ytkA (2)*		Unknown

Only genes with *p*-value < 0.05 are shown, and RNAs with unknown functions have not been included in the table. Only genes with *p*-value < 0.05 are shown. The fold-change (FC) is shown using a linear scale and not as log_2_. FC of the 3 h sample are also listed. Genes in an operon are clustered. In these cases the maximum and minimum FC are indicated. In the operon column the number of genes of the operon is listed between brackets. The regulon column suggests the possible regulator. The percentage shows the probability of regulation indicated by the arrow (up is activation and down is repression). A gene can be part of different regulons, however, only the regulon with the largest percentage of significantly expressed genes is shown. For some regulons the regulation directionality is not present in the Subtiwiki database. This is indicated by 0% for probability of regulation direction and the absence of an arrow

An initial analysis of the genes in Tables 1 and 2 did not reveal clear secretion bottlenecks, such as the induction of the membrane-anchored protein quality control proteases HtrA/B. The only secretion-related effect was a strong downregulation of *scr*, the 4.5 S RNA component of the signal recognition particle SRP, in the 6 h time sample (Table 2) [31]. Expression of *ffh*, coding for the protein subunit of SRP, was not significantly affected (Additional file 1: Table S1). It is reasonable to assume that XynA, like most secreted proteins, uses the SecA-dependent secretion route instead of the co-translational SRP pathway, since SRP is primarily used for the insertion of transmembrane proteins into the cell membrane [31]. When we increased the expression of *scr* by integrating an extra copy driven by the strong xylose-inducible *Pxyl* promoter [32], the XynA levels in the medium did not increase (Additional file 3: Fig. S1), indicating that the reduced levels of *scr* are not limiting XynA secretion.

### Analysis of functional categories

**Fig. 3:**
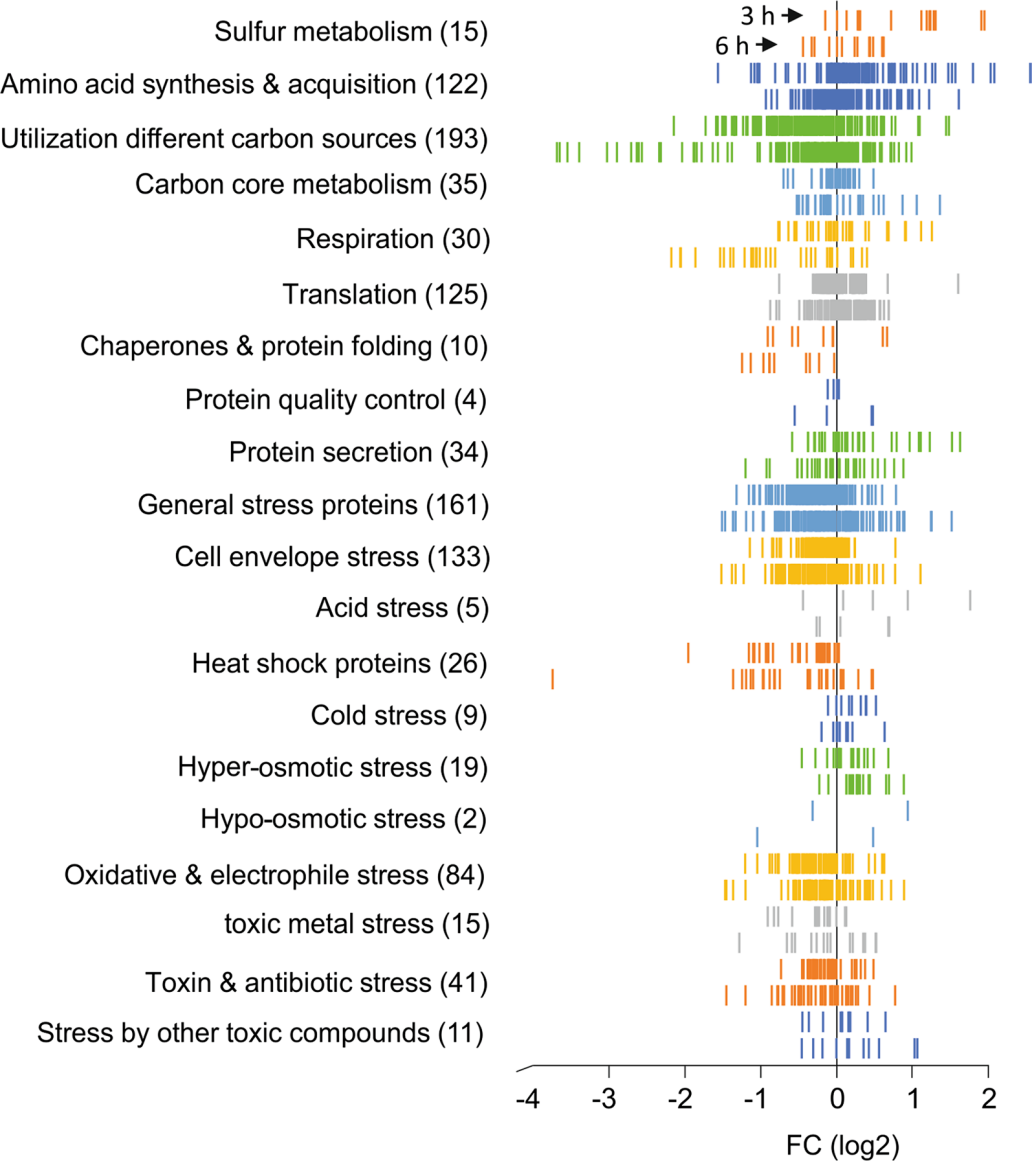
Functional category distribution plot. Log_2_ fold-change values (FC) of genes are plotted against the *x*-axis. The number of genes in each functional category is indicated between brackets. The upper and lower set of bars shown for each category represent the 3 h and 6 h sample, respectively, as indicated by black arrows for the Sulfur metabolism category

Threshold values used to choose the most relevant differential expressed genes are arbitrary. As a consequence, useful gene regulation information can be lost. It can therefore be helpful to analyse transcriptome data using gene set enrichment analysis, based on functional category and/or regulon information, without prior selection of genes using cut-off values. To facilitate such analysis, we developed the Excel add-in GINtool [27], and used functional categories and regulon information that has been collected over time in the main *B. subtilis* knowledge database Subtiwiki [27, 33]. First, we focussed on the functional categories related to stress, protein translation and protein homeostasis, and based on the genes in Tables 1 and 2, also included the functional categories related to respiration, sulfur and carbon metabolism, and amino acid utilisation. As shown in Fig. 3, there are substantial more amino acid synthesis and acquisition genes upregulated in the 3 h time point than are listed in Table 1. Also the downregulation of respiration genes in the 6 h sample is more obvious, compared to the information in Table 2. Interestingly, for both time points the functional category “chaperones and protein folding” is downregulated. This category comprises the conserved protein chaperones GroEL/ES, DnaJ/K and others, but their related genes do not show up in Tables 1 and 2 due to the chosen cutoff for fold change. The "general stress proteins" category, comprising 161 Sigma-B controlled genes, does not seem to be activated in the XynA overexpressing cells, but the "heat shock proteins" appear to be collectively downregulated in both samples. Several genes in the functional category “protein secretion” are slightly upregulated in the 3 h sample. This category is a collection of genes that are involved a wide variety of processes including general secretion, such as the Sec and signal peptidase encoding genes, but also genes encoding the Tat secretion system and secretion processes during sporulation. In fact, the most strongly (2- to 3-fold) induced genes in this category are those form the sporulation operon *spoIIIAB*, involved in the activation of the sporulation sigma factor Sigma-G (Additional file 1: Table S1) [34, 35].

### Analysis of regulon activities

Often functional categories comprise genes that are controlled by different regulators. For example the downregulated functional category "heat shock proteins" include 27 genes comprising several regulons, including the general heat stress genes regulated by the sigma factor SigI, the CssR controlled membrane-anchored protein quality control protease genes *htrA/B*, the Clp AAA + unfoldases encoding genes regulated by CtsR, and the HrcA regulated GroEL/ES and DnaJ/K encoding genes [33]. Therefore, to gain a better understanding what regulation underlies the observed transcription differences, it is useful to focus on regulons. So far, 218 regulons have been identified for *B. subtilis* [33]. When these regulons were sorted, based on their average fold change values, a clear gradation in activities was observed (Additional file 3: Fig. S2), presenting a more natural view of the differential expression than the binary, relevant/non-relevant, distribution based on arbitrary fold change and *p*-value threshold levels.

**Fig. 4:**
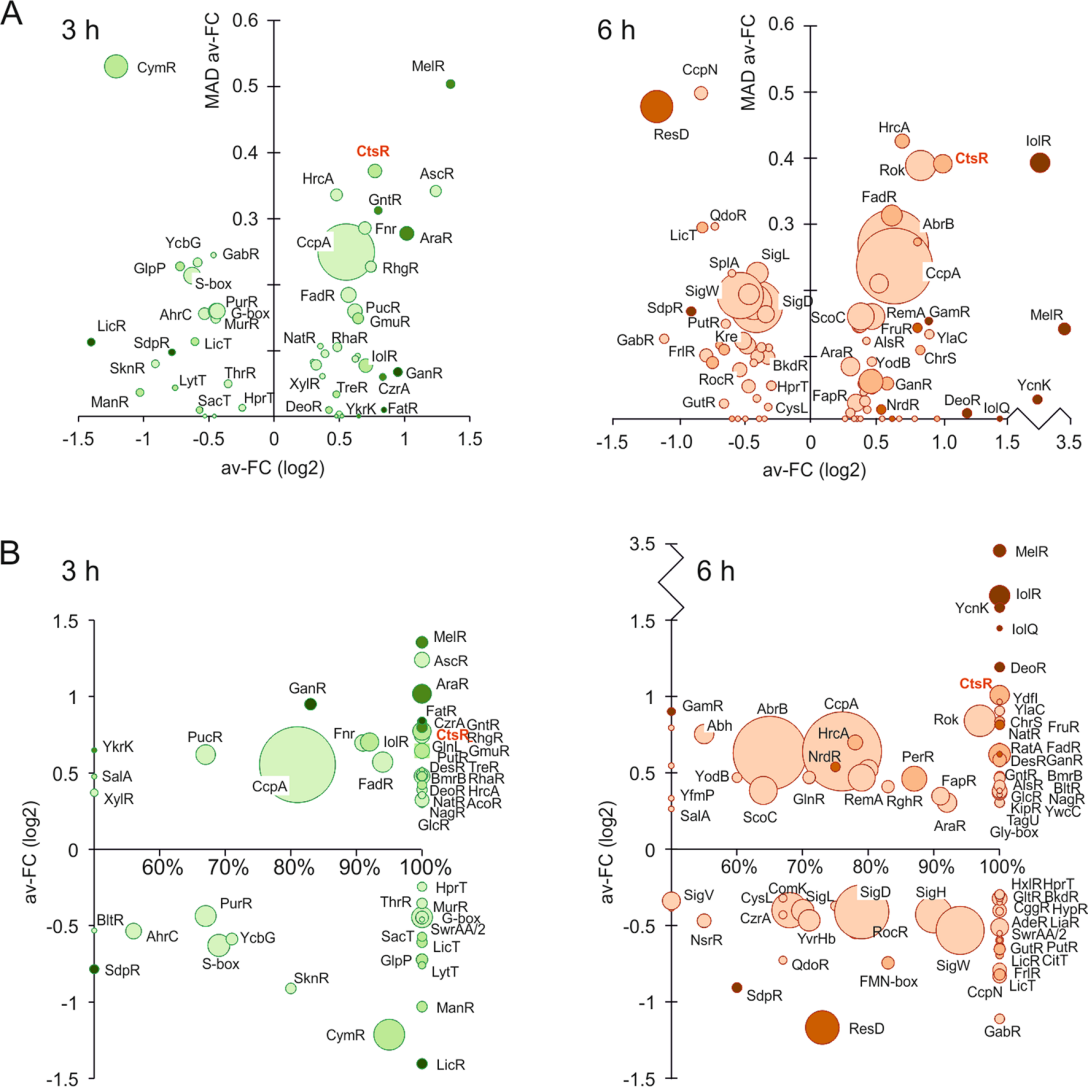
Regulon bubble plots. **A** Average fold changes (av-FC) are plotted against the mean absolute deviation of the fold change (MAD av-FC) for the 3 h (left) and 6 h (right) samples. **B** Average fold-changes plotted against the fraction of regulon genes corresponding to the most likely regulation for the 3 h (left) and 6 h (right) samples. Bubble size indicates the number of regulon genes. Colour intensities indicate average *p*-values of the genes in the regulon classified from dark to light according to average *p*-values of < 0.0625, < 0.125, < 0.25, < 0.5. For clarity, regulons with average *p*-values ≥ 0.5 are not shown. Of note, the average fold changes, MAD, *p*-values and bubble sizes are based on the regulon genes that correspond to the most likely activity state of the regulator. This state, being either activated or repressed, is based on the largest fraction of regulon genes that show a fold change corresponding to one of these two activity states. The CtsR regulon is indicated in red

GINtool can plot the average fold change of regulons against the mean absolute deviation (MAD) of the fold change [27]. The MAD values indicate how uniform the expression differences of the genes in a regulon are and facilitates the visually presentation of regulon activities (Fig. 4a). The GINtool analysis results for all regulons is listed in Additional file 2: Table S2. The largest regulon affected by XynA overexpression in both the 3 h and 6 h samples is the CcpA regulon. CcpA is the main catabolite repressor of *B. subtilis* [36]. The next largest regulon is the AbrB regulon that appears in the 6 h time sample, which is a key transcriptional regulator of transition state genes that become active in the stationary phase [37]. In Fig. 4a these regulons have a positive average fold change value, which means that CcpA and AbrB are more activated when XynA is overexpressed. However, and importantly, since these regulators function primarily as transcriptional repressors, many genes of these regulons are in fact down-regulated upon XynA overexpression. The reason that GINtool assigns a positive sign for the average fold change of these regulon is that the programme takes into account the directionality of regulation (activation/repression) by a regulator when this information is available [27]. This information is crucial to calculate a relevant average fold change, and for *B. subtilis* this information can also be found in the Subtiwiki database [33]. The reason why the directionality of regulation is important is that regulators can simultaneously function as a repressor of some genes and an activator of other genes. For example, the *B. subtilis* response regulator Spo0A represses 104 genes and activates 63 [33]. This means that just averaging the fold change of the genes of a regulon does not give an accurate approximation of the regulator activity.

GINtool estimates the likelihood of activation or repression by calculating the fraction of regulon genes that exhibit a fold change corresponding to the most likely activity state of the regulator [27]. As shown in Fig. 4b, there are many regulons that show a consistent (100%) activity change upon overexpression of XynA. Most of these regulons are related to sugar metabolism (Additional file 2: Table S2), and it is not immediately apparent how these processes could hamper the production of XynA. However, there was one interesting regulon in this list, the CtsR regulon, which comprises, aside of its own gene, the AAA + unfoldase encoding genes *clpE*, *clpC* and *clpX*, and the related protease subunit encoding *clpP* gene. Normally, the expression of these proteins is associated with the presence of misfolded proteins that occurs e.g. after heat shock [38]. However, strong overexpression of proteins can also lead to an increased level of misfolded proteins, and it was therefore surprising that the *clp* genes were collectively downregulated upon XynA overexpression.

### Uncoupling Clp expression from CtsR regulation increases XynA levels

**Fig. 5:**
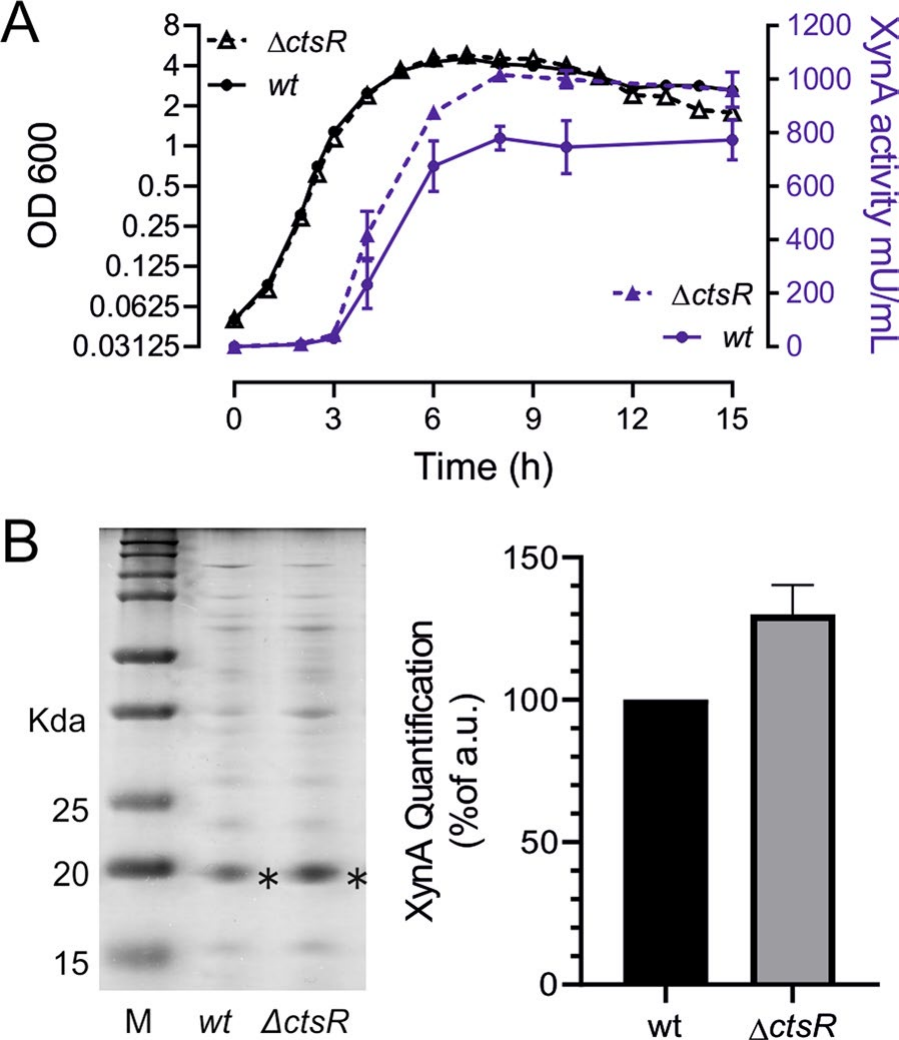
XynA production in a ∆*ctsR* deletion strain. **A** XynA production by the BWB09/pCS58 wild type (wt) and SGB03/pCS58 ∆*ctsR* deletion strains during growth. Averages and standard deviations of 3 independent experiments are shown. **B** Coomassie staining of proteins precipitated from 10 h supernatant samples. Molecular weights are indicated on the left side. The asterisk marks the XynA protein bands in the supernatant samples. Bars on the left represent protein densitometry quantification of the Coomassie-stained gel of three replicates

CtsR functions as a transcriptional repressor and its concentration is controlled by regulated proteolysis. The adaptor McsB kinase specifically binds and targets CtsR for degradation by the ClpCP protease complex [39, 40]. Heat shock or oxidation stress affects the DNA binding activity of CtsR and triggers the activation of McsB [41–43]. Presumably, under XynA overproduction conditions, the ClpCP system is busy dealing with unfolded or misfolded XynA and has less time to engage in CtsR proteolysis. This will then result in increasing levels of CtsR and subsequent down regulation of *clpCP* expression, thus further reinforcing this negative feedback loop. This may explain why we observe a downregulation of the CtsR regulon. It is reasonable to assume that increased levels of Clp chaperones will reduce the level of misfolded proteins, which may help to increase XynA production. To test this, we constructed a *ctsR* deletion strain and measured the production of XynA. As shown in Fig. 5, the ∆*ctsR* mutant showed a normal growth rate, however XynA secretion was approximately 25 ± 5% higher compared to the wildtype strain. In conclusion, induction of the CtsR regulon by removing the CtsR repressor improves the production of XynA.

## Discussion

Here we found that overexpression of XynA in *B. subtilis* downregulates the CtsR regulon. This result did not show up in an earlier published transcriptome study of XynA overexpressing *B. subtilis* [18]. When we compared the data from this study with ours, by selecting all genes that showed a 2 or more fold change in expression with *p*-values < 0.05, we found only 9 genes that showed a comparable expression difference (Additional file 3: Fig. S3). These included the upregulation of the isoleucine biosynthesis genes *ilvB* and *dppB*, and the downregulation of genes in the melibiose utilization *melR* operon and the *ctaF* gene encoding cytochrome-c oxidase subunit IV. In the other study an upregulation of the membrane associated protein quality control proteases HtrA/B and the chaperones GroEL/ES was observed [18], which we did not observe. However, these differences and the lack of overlap in regulated genes can be explained by the distinct experimental conditions used. In the previous study the expression of XynA was induced for only 30 min at the end of logarithmic growth after which RNA samples were taken, whereas in our system XynA was expressed continuously from the constitutively active and strong *amyQ* promoter [30].

In our study we found only one direct secretion related signature and that was the significant drop in *scr* levels, the RNA component of SRP. For *E. coli* it has been shown that reduced *scr* concentrations lead to reduced stability of Ffh, the protein component of SRP, and impaired SRP-dependent translocation [44, 45]. It is unlikely that XynA uses SRP for secretion, as the secretion of proteins into the medium generally relies on the SecA route [46, 47], but since both the SRP and SecA route use the same Sec translocation complex, an impaired SRP system might indirectly affect optimal XynA secretion. However, when we expressed an extra copy of *scr* ectopically from a strong xylose-inducible promoter no improvement in XynA production was observed. Although, we do not have an explanation for why the *scr* concentration goes down in XynA overexpressing cells, it is clear that lower *scr* levels are not limiting XynA production.

High production of XynA by *B. subtilis* affects the activity of a wide variety of regulons, including respiration, copper uptake, toxin protection, sugar utilisation and sporulation regulons. None of these regulons can be functionally linked to either xylanases or secretion stress. Maybe some of the related regulators are also controlled by regulated proteolysis and therefore more active under high XynA production conditions. Of note, the early induction of sporulation regulons did not lead to an early sporulation phenotype (data not shown).

We assume that the increased production of XynA in a ∆*ctsR* mutant is caused by the increased levels of the ClpC/E/X chaperones and/or by the protein quality protease complexes they can form together with ClpP. There are several examples of improved production by inducing the expression of chaperones. For example, increasing the expression of trigger factor together with GroEL/ES increases the production of human protein ORP150 and human lysozyme in *E. coli* [48]. Increasing the levels of GroEL/ES together with DnaJ/K-GrpE has been shown to improve the production of *Bacillus stearothermophilus* α-amylase by *B. subtilis* [49]. Interestingly, the *groEL/ES* and *dnaJ*/*K* genes are controlled by the transcriptional repressor HrcA [50, 51], and this protein is also controlled by proteolytic degradation by ClpP [52], and in fact we do see a downregulation of HrcA regulon genes in our analysis (Fig. 4). So it is possible that inactivation of CtsR improves production in different ways, including an indirect increase in *groEL/ES* and *dnaJ/K* expression. It is also important to keep in mind that CtsR regulates other genes, such as *disA* and *radA* involved in DNA repair and recombination, the ATP-dependent protein quality control protease encoding *lonA* gene, and the genes *ysxC* and *trxA*, involved in ribosome assembly and oxidative damage protection, respectively [53, 54]. Therefore, future studies will be necessary to determine whether the improved XynA production in a ∆*ctsR* strain is directly caused by the protein chaperone and quality control activities of the Clp proteins. However, due to the many regulatory roles of these proteins, it will be difficult to obtain unambiguous evidence for this.

## Conclusions

Here we show that an advanced gene set enrichment analysis can reveal useful information that would have been missed in case subjective threshold values would have been used to select interesting genes. Our analysis indicates that increased expression of the CtsR regulon, by removing the related transcriptional repressor, leads to increased production of XynA. Due to the feedback control of CtsR by ClpCP it is reasonable to assume that strong overproduction of other proteins in *B. subtilis* will also lead to a reduction of *clp* gene expression, therefore inactivation of CtsR might also improve production levels of other proteins in this organism. In fact, this approach might work in many production organisms since both the Clp proteins and CtsR are conserved in firmicutes.

## Methods

### Bacterial strains and growth conditions

Bacterial strains and plasmids used in this study are listed in Additional file 3: Table S3. The wild type tryptophan prototrophic *B. subtilis* 168 derivative BSB1 was used for expression. Nutrient Luria–Bertani medium (LB, containing 10 g/L Tryptone, 5 g/L Yeast Extract, 10 g/L NaCl) was used for general growth of both *B. subtilis* and *E. coli.* Supplements were added as required: erythromycin (5 µg/mL), kanamycin (50 μg/mL), spectinomycin (150 µg/mL), ampicillin (100 µg/mL), and Xylose (Xyl, 0.5% or 2%, w/v). For *B. subtilis* XynA secretion profile measurements, strains were inoculated and grown overnight at 30 °C to prevent sporulation. The next morning, the overnight culture was diluted into fresh and prewarmed medium to a start OD_600_ of 0.05. The culture was grown at 210 rpm shaking (37 °C) and sampled at desired timepoints for follow-up enzymatic or protein experiments. We used 100 mL Erlenmeyer flasks for 10 mL cultures and 250 mL Erlenmeyer flasks for 25 mL cultures to guarantee proper aeration. The medium was supplemented with 50 μg/mL kanamycin to maintain the plasmids.

For *B. subtilis* DNA transformation, the Spizizen-starvation media (SMM, containing 15 mM (NH_4_)_2_SO_4_, 80 mM K_2_HPO_4_, 44 mM KH_2_PO_4_, 3 mM tri-sodium citrate, 0.5% glucose, 6 mM MgSO_4_, 0.2 mg/mL tryptophan, 0.02% casamino acids, and 0.00011% ferric ammonium citrate (NH_4_)_5_Fe(C_6_H_4_O_7_)_2_) were used, as described before [55].

### Plasmid and mutant construction

All primers used for cloning are listed in Additional file 3: Table S4, and all constructs were sequenced to omit possible mutations. The construction of BWB09 (*trpC*+, ∆*xynA,* ∆*amyE*). was described in [56]. Plasmid pCS58 [27] for XynA overproduction was transformed into BWB09. Plasmid pCS58 was used as template to amplify the vector backbone sequence omitting the *xynA* ORF. This plasmid, pBW17 [27], was used as negative control.

Plasmid pBW18 used for *scr* overexpression was constructed from plasmid pHJS103 [57]. The xylose-inducible promoter *Pxyl* and *amyE*-integration flanking sequences were amplified from pHJS103, and *scr* sequence containing S17-5’UTR was amplified from BSB1 chromosomal DNA. Subsequently, both PCR products were ligated by Gibson assembly [58] and transformed into *E. coli* TOP10 cells [59]. pBW18 was then isolated, linearized by restriction enzyme digestion, and transformed into competent BWB06 cells, resulting in *scr* overexpression strain SGB01.

The *ctsR* mutant SGB03 was constructed by transformation of chromosomal DNA from a ∆*ctsR* mutant, obtained from the BKE *B. subtilis* genome-scale deletion library [60], into competent BWB09 cells.

### RNA extraction for RNA-seq

RNA extraction was based on the methods described in [61, 62]. Briefly, 2 mL cells were collected from the logarithmic growth phase (3 h) and stationary growth phase (6 h). Cell pellets were resuspended in 0.4 mL ice-cold growth medium and added to a screw cap tube containing 1.5 g glass beads (0.1 mm), 0.4 mL phenol/chloroform/isoamyl alcohol mixture (25:24:1) and 50 μL 10% SDS, vortexed to mix, and stored by flash freezing in liquid nitrogen. Cell disruption was achieved by bead beating (Precellys 24). After centrifugation, RNA in the upper aqueous phase was ethanol-precipitated, washed twice with 70% ice cold ethanol, dried and dissolved in water. DNA was removed by DNAseI (NEB) treatment. The RNA was then extracted by a second-round of P/C/I extraction, followed by ethanol precipitation and 70% ethanol washing, and dissolving in water.

### RNA-seq and sequencing data analysis

Prior to deep-sequencing, the RNA samples were treated with the MICROBExpress™ Bacterial mRNA Enrichment Kit (Thermo Fisher) to remove most of the 16S and 23S rRNA. Subsequently, the RNA-seq libraries were constructed using the NEBNext^®^ Ultra™ II Directional RNA Library Prep Kit from Illumina^®^ (New England Biolabs) using NEBNext^®^ Multiplex Oligos for Illumina^®^ (New England Biolabs), according to the manufacturer's protocol. Sequencing was performed on an Illumina NextSeq 550 System using NextSeq 500/550 High Output v2.5 kit (75-bp read length), and the raw data were processed using the web-based platform Galaxy. We aimed at a sequencing depth of 5–10 million reads per library [28]. *Trimmomatic* was used to trim the adaptor sequence and filter bad reads [63]. The trimmed reads were aligned to the *Bacillus* reference genome (NC_000913) with *Bowtie2* [64]. After mapping, aligned reads were counted using *FeatureCount* [65]. *Deseq2* was used to determine differentially expressed features between samples [66]. The in-house developed software tool, GINtool, was used to analyse the transcriptome data [27], using prior knowledge on operons, functional categories and regulons obtained from the Subtiwiki database [33].

### XynA enzyme activity assay

100 μL cells were taken from the culture transferred into a 1.5 mL Eppendorf tube and centrifuged at 20,000 RCF for 1 min at 4 °C, and then 70 μL supernatant was transferred to a new tube and stored by flash freezing in liquid nitrogen and storage at − 80 °C. XynA enzyme activity in the supernatant was determined using the fluorescence based assay EnzChek^®^ Ultra Xylanase Assay Kit (Thermo Fisher Scientific), according to the manufacturer’s instructions.

### SDS-PAGE electrophoresis and Coomassie staining

1 mL culture was transferred to a 1.5 mL Eppendorf tube and centrifuged at 20,000 RCF for 1 min at 4 °C, and 800 μL supernatant was carefully transferred to a new tube containing 200 μL ice-cold 100% (w/v) Trichloroacetic acid (TCA), vortexed to mix and precipitated at − 20 °C overnight. The sample was centrifuged at 20,000 RCF for 20 min at 4 °C, and the protein pellet was washed with 800 μL ice-cold acetone and air dried. The protein pellet was resuspended in 1X SDS-PAGE loading buffer containing 1 mM PMSF and Protease Inhibitor Cocktail (Sigma-Aldrich). A 5 min bath sonication step was used to quickly dissolve the pellet. All samples were denatured at 95 °C for 5 min and separated on a 14% SDS-PAGE gel. After electrophoresis, the gel was stained with Colloidal Coomassie according to Bio-Rad's protocol. Sample loading amount was normalized based on optical density of the culture_,_ and an amount of cells corresponding to an OD_600_ of 0.2 was loaded per lane. Images were taken by Odyssey Fc Imaging System and analysed with Empiria Studio software (LI-COR Biosciences).

### Supplementary Information

**Additional file 1.** **Table S1.** Normalized reads, log2FoldChange and P values of all transcripts of 3 h and 6 h samples.

**Additional file 2.** **Table S2.** The GINtool analysis results for all regulons of 3 h and 6 h samples.

**Additional file 3.** Supplementary information.

## Data Availability

RNA-seq data have been submitted to and are accessible in the Gene Expression Omnibus (GEO), accession number GSE217916. The GINtool source and executable can be downloaded from GitHub (ScienceParkStudyGroup/GINtool: Gene Information Network add-in for Excel).
