# Supplementary material for: Induction of the CtsR regulon improves Xylanase production in Bacillus subtilis
Source: Microb Cell Fact. 2023 Nov 9;22:231. doi: 10.1186/s12934-023-02239-3 (PMC10633939; doi:10.1186/s12934-023-02239-3)
Supplement: Supplementary file 3 — Additional file 3. Supplementary information. [file 12934_2023_2239_MOESM3_ESM.pdf]

Supplementary Information

Induction of the CtsR regulon improves Xylanase production in *Bacillus subtilis*

Biwen Wang, Frans van der Kloet, Leendert Hamoen

Table of content

Table S3 Strains and plasmids used in this study

Table S4 Primer sequences used in this study

Fig. S1 XynA production profile in an *scr* overexpressing strain

Fig. S2 Regulon fold-change spread plot of the 3 h and 6 h samples

Fig. S3 Comparison of our data with a previous XynA transcriptome study

Table S3. Strains and plasmids used in this study

Strain	Genome type	Source
BSB1	*B. subtilis wildtype* 168 trp+;	Lab storage
BWB06	BSB1 trp+; Δ*xynA*	[2]
BWB09	BSB1 trp+; Δ*xynA*, Δ*amyE*	[2]
BKE00830	1A1 Δ*ctsR::ery*	[3]
SGB01	BSB1 trp+; Δ*xynA*; *amyE::*P_{xyl}*-scr*	This study
SGB03	BSB1 trp+; Δ*xynA*, Δ*amyE*, Δ*ctsR::ery*	This study
**Plasmid**		
pCS58	P_{amyQ}*-xynA, bleo(Km)*	DSM
pBW17	P_{amyQ}*-empty, bleo(Km)*	This study
pHJS103	*amyE_Down-*P_{xyl}*-sfGFP-spec-amyE_Up, Sp, Ap*	[4]
pBW18	*amyE_Down-*P_{xyl}*-scr-spec-amyE_Up, Sp, Ap*	This study

Table S4. Primers used in this study

Name	Sequence (5'-3')	Target
BW05	CTAATTGAGAGAAGTTTCTATAGAATTTT	SpR-mazF-Fw
BW06	CTACCCAATCAGTACGTTAATTT	SpR-mazF-Rv
BW34	AAAGGAGCGATTTACATATGTAACAGATCATCCTTAATCA	pEmpty1-Fw
BW35	TGATTAAGGATGATCTGTTACATATGTAAATCGCTCCTTT	pEmpty1-Rv
BW41	CAGATCATCCTTAATCAGGGGTAGCTAACG	XnyAUp-Fw
BW42	GAAACTTCTCTCAATTAGATTTCATGTAAACCGAGAACCA	XnyAUp-Rv
BW45	GCAAAAGCCCTTATGAGGGCTTTTTTAATTGTTGTTTGCAGTAAC	XnyADn-Fw
BW46	ACCCCTGATTAAGGATGATCTGATGTTACCTCCTATAATATTTTTTCCG	XnyADn-Rv
BW47	AATTAACGTACTGATTGGGTAGTTCTTAGTTGGATTATCGGCAGC	XnyA-Fw
BW48	GGATGATCTGTTACCACACTGTTACGTTAGAACTTCCACTAC	XnyA-Rv
BW49	ATGATCAATTGGGGGCCGTTTTAACGATTGCTGCC	AmyEUp-Fw
BW50	TCCCGTCTAGCCTTGCCCTCTTGACACTCCTTATTTGA	AmyEUp-Rv
BW51	GGGCAAGGCTAGACGGGACTTACCGAAAGAAA	AmyEDn-Fw
BW52	TATAGAAACTTCTCTCAATTAGCCCGCTCTTTTGGCAGGCCGC	AmyEDn-Rv
BW53	AACGTACTGATTGGGTAGGCCATTCAGACATCTCCGA	AmyE-Fw
BW54	CAGACCTGGCATTGATCGTGCCTGTCAGTTTAC	AmyE-Rv
BW60	CCTAGGAATCTCCTTTCTAGATG	pHJS103-Fw
BW61	AAAATGTCCAGACTTCGGA	pHJS103-Rv
BW131	CATCTAGAAAGGAGATTCCTAGGGCATCGTAATAGATGCAACATA	Scr-Fw
BW132	TGGATCCGAAGTCTGGACATTTTCTAATATTAGGTGAGTCTGAAAAT	Scr-Rv
TerS385	CCCGAGGTGCGGACGGGCGCCATGA	AmyE-Ex-Fw
TerS386	GGCTCAGCGGGATCAGGCGCTGCGCAA	AmyE-Ex-Rv

Fig. S1.

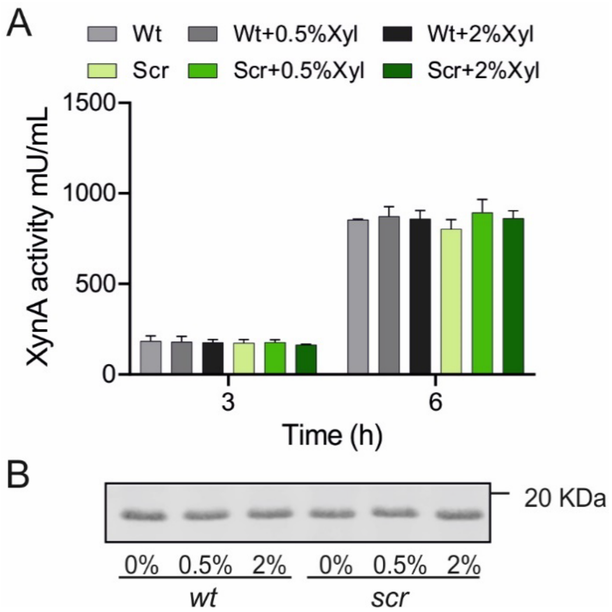

Fig. S1. XynA production profile in an *scr* overexpressing strain

(A) XynA enzyme activity in the supernatant of BWB09/pCS58 (*wt*) and *scr* overexpressing strain SGB01/pCS58 (*Scr*) at 3 h and 6 h growth in LB supplemented with 0 %, 0.5 % and 2 % xylose. (B) Coomassie-stained gel shows XynA band precipitated from 6 h supernatant samples. Samples were normalized by cell density. Three independent replicates were performed to confirm the reproducibility of results.

Fig. S2.

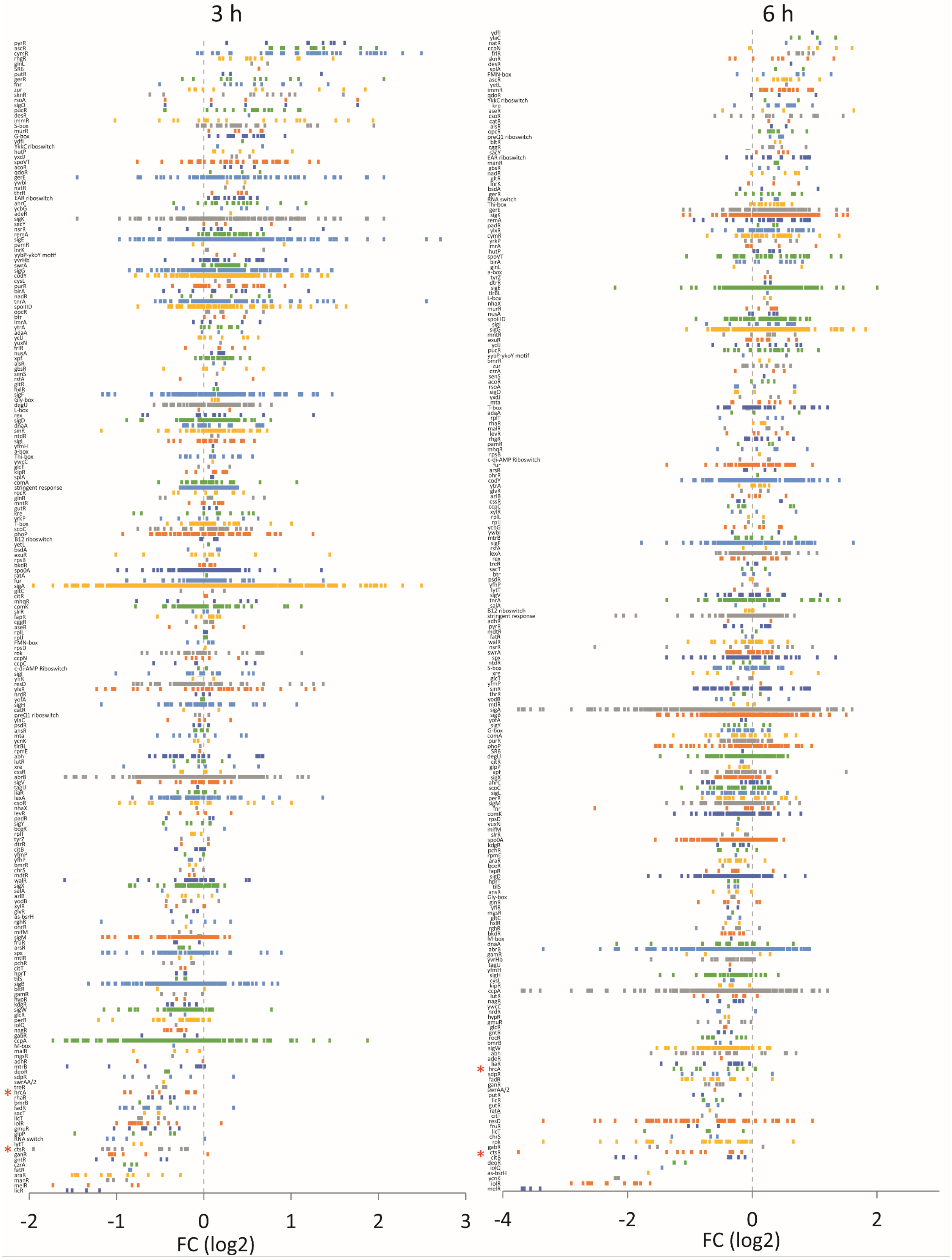

Fig. S2. Regulon fold-change spread plot of the 3 h and 6 h samples
Log_2 fold-change values (FC) of regulon genes were plotted along the X-axis. Regulons were sorted according to the average of the fold-change of all genes in a regulon. The CtsR and HrcA regulons are marked with red asterisks.

Fig. S3.

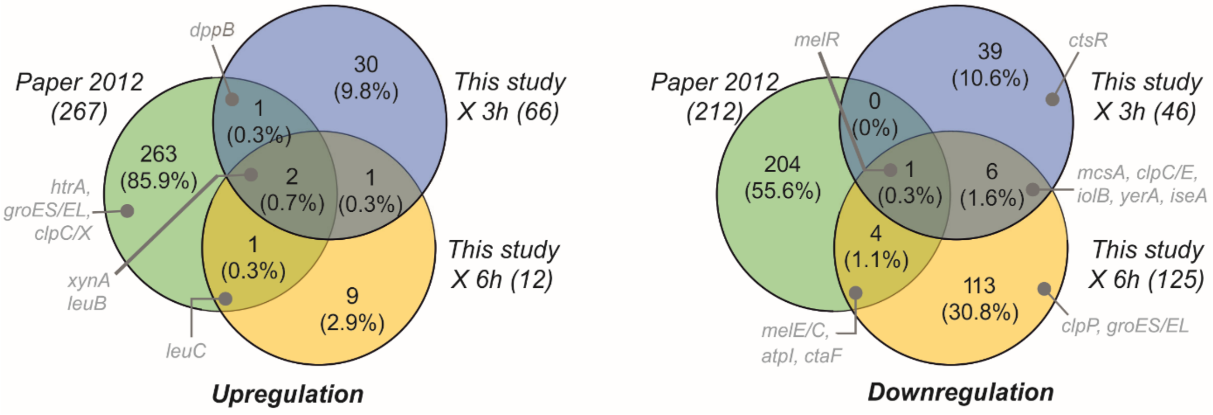

Fig. S3. Comparison of our data with a previous XynA transcriptome study

A significance threshold of ≥ 2 fold change with p-value < 0.05 was used to compare our data with a previous XynA transcriptome study [1].

References

1. Marciniak BC, Trip H, van-der Veek PJ, Kuipers OP. Comparative transcriptional analysis of *Bacillus subtilis* cells overproducing either secreted proteins, lipoproteins or membrane proteins. Microbial Cell Factories. 2012;11:66.

2. Schäfer A-B, Steenhuis M, Jim KK, Neef J, O'Keefe S, Whitehead RC, et al. Dual Action of Eeyarestatin 24 on Sec-Dependent Protein Secretion and Bacterial DNA. ACS Infect Dis. American Chemical Society; 2023;9:253–69.

3. Koo B-M, Kritikos G, Farelli JD, Todor H, Tong K, Kimsey H, et al. Construction and analysis of two genome-scale deletion libraries for *Bacillus subtilis*. Cell systems. Elsevier; 2017;4:291–305.

4. Müller A, Wenzel M, Strahl H, Grein F, Saaki TNV V, Kohl B, et al. Daptomycin inhibits cell envelope synthesis by interfering with fluid membrane microdomains. Proceedings of the National Academy of Sciences of the United States of America. National Acad Sciences; 2016;113:E7077–86.
